# Assessing Fermentation Broth Quality of Pineapple Vinegar Production with a Near-Infrared Fiber-Optic Probe Coupled with Stability Competitive Adaptive Reweighted Sampling

**DOI:** 10.3390/molecules28176239

**Published:** 2023-08-25

**Authors:** Sumaporn Kasemsumran, Antika Boondaeng, Sunee Jungtheerapanich, Kraireuk Ngowsuwan, Waraporn Apiwatanapiwat, Phornphimon Janchai, Pilanee Vaithanomsat

**Affiliations:** 1 Laboratory of Non-Destructive Quality Evaluation of Commodities, Kasetsart Agricultural and Agro-Industrial Product Improvement Institute (KAPI), Kasetsart University, Bangkok 10900, Thailand; aapsnj@ku.ac.th (S.J.); aapkrn@ku.ac.th (K.N.); 2 Laboratory of Enzyme and Microbiology, KAPI, Kasetsart University, Bangkok 10900, Thailand; aapakb@ku.ac.th (A.B.); aapwpa@ku.ac.th (W.A.); aappmj@ku.ac.th (P.J.); aappln@ku.ac.th (P.V.)

**Keywords:** near-infrared, fiber-optic probe, stability competitive adaptive reweighted sampling, pineapple vinegar, fermentation

## Abstract

In this study, the performance of a near-infrared (NIR) fiber-optic probe coupled with stability competitive adaptive reweighted sampling (SCARS) was investigated for the analysis of acetic acid, ethanol, total soluble solids, caffeic acid, gallic acid, and tannic acid in the broth of pineapple vinegar during fermentation. The NIR spectra of the broth samples in the region of 11,536–3956 cm^−1^ were collected during vinegar fermentation promoted by *Acetobacter aceti*. This continuous biological process led to changes in the concentrations of all analytes studied. SCARS provided optimized and stabilized NIR spectral variables for the construction of a partial least squares (PLS) model for each analyte using a small number of optimal variables (under 88 variables). The SCARS-PLS model outperformed the conventional PLS model, and achieved excellent accuracy in accordance with ISO 12099:2017 for the four prediction models of acetic acid, ethanol, caffeic acid, and gallic acid, with root-mean-square error of prediction values of 0.137%, 0.178%, 0.637 μg/mL and 0.640 μg/mL, respectively. In contrast, only an acetic acid content prediction model constructed via the conventional PLS method and using the whole spectral region (949 variables) could pass with acceptable accuracy. These results indicate that the NIR optical probe coupled with SCARS is an appropriate method for the continuous monitoring of multianalytes during vinegar fermentation, particularly acetic acid and ethanol contents, which are indicators of the finished fermentation of pineapple vinegar.

## 1. Introduction

Vinegar is a food product that is produced via double fermentation, in which an alcohol is produced via the fermentation of sugars by yeast, followed by the degradation of the alcohol to acetic acid under oxygenated conditions with bacteria of the genus Acetobacter [1,2]. The consumption of fermented vinegar is associated with many benefits, including the regulation of blood sugar levels [3], reduced cholesterol during regular consumption [4], increased liver efficiency owing to the conversion of acids in the citric acid cycle, and improved calcium absorption [5]. Fermented vinegar is produced from various carbohydrate-based raw materials. Grapes are the most common raw material used in vinegar production worldwide. Rice is used in the production of traditional alcoholic beverages in China, Japan, and South Korea, and is also a common source of vinegar in these countries. Thailand and other Southeast Asian countries produce various fruits suitable for fruit vinegar production, including mango, kaki, berries, mangosteen, dragon fruit, and pineapple [2].

The production of pineapples in Thailand is expected to increase annually owing to their popularity in many provinces of the country. Fresh pineapples are consumed and used in fruit canneries. However, the oversupply of pineapples has increased during the production season owing to an imbalance between supply and demand in both domestic and export markets. The quantity of low-grade pineapples increases in an oversupply situation, reducing the price; therefore, the excess supply must be overcome [6]. Oversupplied pineapples are used to add value to food and beverage products. Processed pineapple products are generally priced better and have a longer shelf life than fresh ones. Pineapple vinegar is an outstanding product of pineapple processing with a naturally sour taste, unique aroma, and golden-brown color. Pineapple vinegar is valuable in the context of healthy probiotic foods and has excellent food preservation properties owing to its rich acetic acid content [1]. These properties of vinegar could promote food producers to use the oversupply of pineapples to produce vinegar, solving the problem of pineapple waste.

Nevertheless, according to Thailand’s Ministry of Public Health (No. 204) B.E., fermented vinegar should have a minimum acetic acid content of 4% and a maximum residual alcohol content of 0.5% [7]. Quality control of fermented vinegar should be performed to ensure the quality and safety of foods. Pineapples are highly nutritious and rich in antioxidants such as vitamin C and phenolic compounds [8]. While the acetic acid and alcohol contents of vinegar products must be determined to ensure compliance with the regulatory requirements, measuring the content of phenolic compounds in fermented pineapple vinegar is also important because these compounds play an important role in antioxidant activity. The analysis of these crucial parameters requires several conventional methods and instruments, including high-performance liquid chromatography (HPLC) for the determination of acetic acid and phenolic compounds, and gas chromatography (GC) for ethanol analysis. Continuously quantifying all relevant parameters during the fermentation of pineapple vinegar is almost impossible owing to the following limitations: (1) conventional analytical methods are typically rather time-consuming, causing delayed results, (2) the unresponsive analysis of real-time samples, and (3) the fact that samples must be collected during the fermentation process for analysis, resulting in sample loss [9,10]. Conventional techniques are therefore unsuitable for rapid detection during fermentation.

Consequently, spectroscopic analysis using the near-infrared (NIR) method is a promising alternative technique for the analysis of vinegar, both in finished products and during the fermentation process. Table 1 shows the literature review of the quantitative analysis of vinegar using NIR spectroscopy. Several studies have reported the use of NIR spectroscopy with cuvettes to determine vinegar quality (Table 1) [11,12,13,14]. The NIR analysis of vinegar samples has also been achieved using a liquid cell coupled to a reflector cover [15,16,17] and vials [18,19,20]. In previous studies, vinegar samples have been collected from packages, bottles, or fermentation systems and placed into sample cells (cuvettes, a liquid cell with a reflector cover, and vials) for NIR analysis. A practical routine for continued bioanalysis typically considers the sampling time and sample loss during the sampling process for microbiological and chemical analyses. Despite its suitable characteristics and convenient analysis of samples during fermentation processes, no previous studies have used an NIR fiber-optic probe for the analysis of pineapple vinegar during fermentation. Additionally, the NIR fiber-optic probe offers real-time measurement without sampling, sample preparation, or repetitive procedures, thereby enabling continuous measurement. The probe has the potential to replace conventional chemical methods for vinegar analysis. Furthermore, most early studies desired an NIR analysis method for commercial vinegar or vinegar supernatants without the effect of light scattering caused by insoluble solids (Table 1). The insoluble solids occur from the metabolites of microbes and are typically presented in samples during vinegar fermentation. Thus, former studies have produced limited findings regarding the use of fiber optical NIR probes, and samples have contained insoluble particles for the quantification of constituents in vinegar during biological processes.

Therefore, a method of monitoring multianalytes during the pineapple vinegar fermentation process using an NIR fiber-optic probe combined with a wavelength selection method named stability competitive adaptive reweighted sampling (SCARS) [21] was used in this study. The system comprising an FT–NIR spectrometer and a liquid probe was assembled in order to perform NIR data measurement and fulfill the former NIR studies. The SCARS–partial least square (PLS) models for the prediction of the acetic acid, ethanol, total soluble solids (TSS), caffeic acid, gallic acid, and tannic acid concentrations in vinegar samples were developed from a small number of selected spectral variables for a specific analyte under optimization and stability testing using SCARS calculations. The validation method was used to prove the model performance by testing the calibration model with the external prediction samples taken from the new fermentation batch.

The objective of this study was to develop optimized PLS models of acetic acid, ethanol, TSS, caffeic acid, gallic acid, and tannic acid for NIR fiber-optic probe measurements during vinegar fermentation. The optimized NIR spectral variables for these compounds were determined using SCARS. The performance of the SCARS–PLS models developed using the optimized variables was compared with that of conventional PLS models according to the criteria of ISO 12099:2017 [22] for NIR analysis. Therefore, the feasibility of using an optical NIR probe coupled with the SCARS technique was investigated for the continuous evaluation of samples during vinegar fermentation.

## 2. Results and Discussion

### 2.1. Chemical Change in Vinegar Fermentation

**Figure 1:**
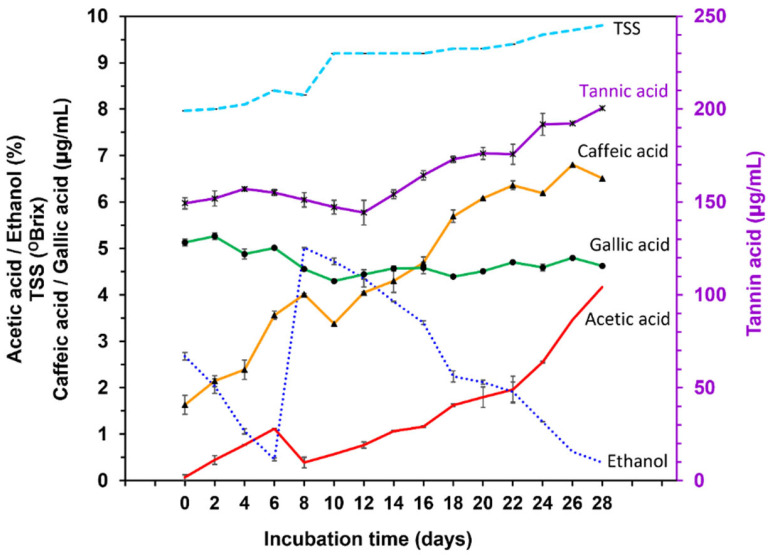
The evolution in the acetic acid (red solid line), ethanol (midnight blue dot line), TSS (blue dash line), caffeic acid (orange solid line; ▲), gallic acid (green solid line; ●) and tannic acid (purple solid line; x) contents of pineapple vinegar during simultaneous vinegar fermentation by *A. aceti* TISTR 102; data are expressed as the mean ± SD.

Figure 1 shows the average values obtained from the chemical analysis of one batch of pineapple vinegar during fermentation. Pineapple vinegar was obtained via sequential fermentation using *Saccharomyces cerevisiae* var. Burgundy, and *Acetobacter pasteurianus* TISTR 102. In the first fermentation step, a mixture of sterilized pineapple juice, pineapple wine, and starter culture was fermented for 6 days at 30 °C. The ethanol content of the mixture decreased during this first incubation period, whereas the acetic acid content increased by 1.12 ± 0.10% *w/v* (Figure 1). Acetic acid is produced via the oxidation of the alcohol in the fermentation broth by *A. aceti* TISTR 102 under aerobic conditions [1]. The concentration of acetic acid decreased from 1.12 ± 0.10% *w/v* to 0.39 ± 0.01% *w/v*, while that of ethanol increased from 0.46 ± 0.16% *v/v* to 5.02 ± 0.06% *v/v* after adding pineapple wine to the vinegar fermenter after 6 days (Figure 1), which induces the second step of fermentation. The efficiency of acetic acid production increased continuously owing to the oxidation of ethanol from wine with *A. aceti* TISTR 102 in fermented broths. Finally, an acetic acid concentration above 4% and a residual alcohol concentration of 0.39% were obtained after 28 days of pineapple vinegar fermentation (Figure 1).

The TSS content increased slightly from 7.97 ± 0.05 °Brix to 9.80 ± 0.00 °Brix during acetification production (Figure 1). The changes in TSS during fermentation may have been caused by an oxidizing mechanism between the acetic bacteria and substrates, including alcohols, sugars, sugar alcohols, and acidic sugars in the fermented broths; this is in an oxidative fermentation process involving the oxidation of the substrate at the outer surface of the cell membrane facing the periplasm. The oxidation products were then excreted from the cell and deposited in the juice or vinegar [23], which produced a significant change in the concentration of TSS after 6 days. After this, pineapple wine was added to the fermentation tank, resulting in an increase in TSS from 8.30 ± 0.00 °Brix to 9.20 ± 0.00 °Brix owing to the increased oxidative activity (Figure 1).

The composition of the vinegar obtained from pineapples during sequential fermentation was 4.17 ± 0.02% *w/v* acetic acid, 0.39 ± 0.002% *v/v* residual alcohol, 9.80 ± 0.00 °Brix TSS, 6.51 ± 0.02 µg/mL caffeic acid, 4.62 ± 0.07 µg/mL gallic acid and 200.55 ± 5.93 µg/mL tannic acid. This composition contained acetic acid, and the residual alcohol levels met the specifications of the Ministry of Public Health of Thailand (No. 204) B.E. 2543 [7] and the Food and Drug Administration (FDA) [24], which stipulate that processed vinegar should contain at least 4 g of acetic acid per 100 mL. In Figure 1, the phenolic compounds, including caffeic, gallic, and tannic acid, were found in pineapple vinegar at similar contents to those observed in previous studies. Mohamad et al. [25] reported that the caffeic acid and gallic acid contents in pineapple vinegar were 218.91 ± 3.24 µg/mL and 862.61 ± 4.38 µg/mL, respectively. Chiet et al. [26] observed that the concentration of gallic acid and tannic acid in pineapple juice ranged from 289.41 ± 16.20 µg/mL to 474.84 ± 12.70 µg/mL and from 189.52 ± 4.44 µg/mL to 305.28 ± 8.00 µg/mL, respectively. The pineapple vinegar obtained in our study contained a level of tannic acid (200.55 ± 5.93 µg/mL) similar to that previously reported, while the levels of caffeic acid and gallic acid were relatively low compared to those measured by earlier studies (Figure 1). This difference may have arisen due to the use of degraded pineapple in vinegar production and the dilution of the pineapple juice prior to use. In addition, the amount and type of phenolic compounds in each pineapple vinegar differ depending on the variety, maturity, and quality of the pineapples used as raw materials, and the fermentation processes used in vinegar production.

The evolution of the caffeic acid, gallic acid, and tannic acid concentrations is shown in Figure 1. The caffeic acid and tannic acid contents tended to increase with fermentation time, likely because both phenolic compounds exist in the form of conjugated and related compounds in the pineapple substrates [27,28]. These related compounds may hydrolyze in an acidic environment upon fermentation, thereby increasing the caffeic and tannic acid contents in proportion to the acetic acid content during vinegar production [28]. The gallic acid content obtained during vinegar fermentation was relatively stable throughout the fermentation process because gallic acid is one of the most stable phenolic compounds. Therefore, the amount of gallic acid obtained from the pineapple substrates remained consistent throughout the vinegar fermentation process. The observed quantitative evolution of the three phenolic compounds demonstrates that sequential fermentation can be used to produce pineapple vinegar with the highest amounts of tannic acid and caffeic acid, and slightly reduced amounts of gallic acid.

### 2.2. Statistical Parameters of Acetic Acid, Ethanol, TSS, Caffeic Acid, Gallic Acid and Tannic Acid in Calibration and Prediction Sets for NIR Analysis

The statistical parameters, including the range, mean, standard deviation, and number of samples for each constituent analyzed in the calibration and prediction sets, are shown in Table 2. To demonstrate the efficiency of the NIR probe in combination with the SCARS method, broth samples for the prediction set were selected from one batch of the continuous fermentation with narrow content ranges (Table 2). The contents of the analytes of interest in the prediction set samples were within the ranges of the two fermentation batches of the calibration set.

### 2.3. NIR Spectra of Fermentation Broth in Pineapple Vinegar Production

**Figure 2:**
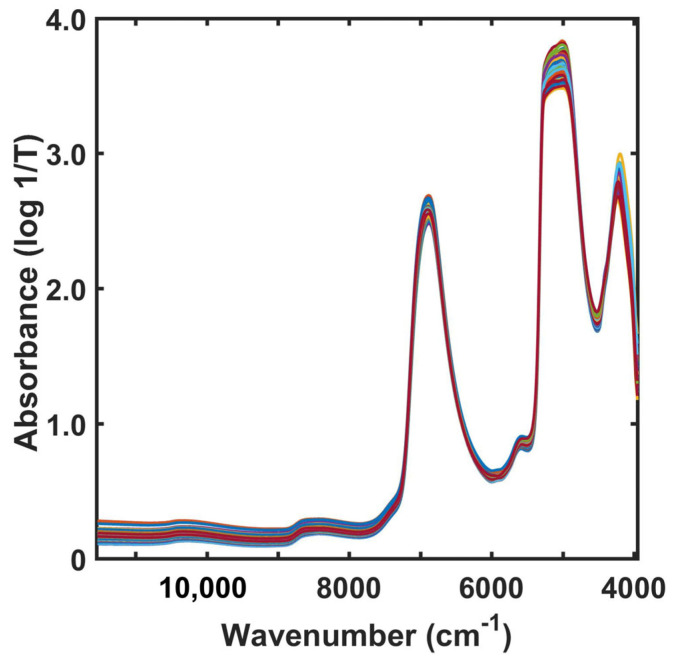
Original NIR absorption spectra of 162 broth samples from pineapple vinegar fermentation (different colors mean different samples).

The original NIR spectra of 162 broth samples obtained from two vinegar fermentation cycles were obtained in the wavenumber region of 11,536–3956 cm^−1^ (Figure 2). The spectra were dominated by two strong absorption bands at approximately 6900 and 5150 cm^−1^. The former band was associated with a combination of the OH symmetric and antisymmetric stretching modes of water, while the latter arose from a combination of the OH stretching and bending vibrations of water. The intensity of the absorption bands indicates that water is the major component of pineapple vinegar.

Furthermore, these bands were ascribed to the COOH and OH groups formed by acetic acid and ethanol, which are minor components of vinegar. Other weak absorption bands centered at approximately 8500, 6000, and 5600 cm^−1^ were assigned to the stretching and deformation vibrations of CH_3_, CH, and OH groups, respectively, which are found in acetic acid, ethanol, sugars, and aromatic polyphenol compounds [29]. Similar results have been reported by other groups [30,31]. The variations in the intensity of the bands corresponding to water and that of the bands in other spectral regions were due to variations in the concentrations of all investigated constituents; however, such variations were not clearly observed in the NIR spectra of the fermented pineapple vinegar. The changes in the NIR absorption spectra of the broth samples during the fermentation process were disordered owing to the light-scattering effect. The NIR fiber-optic probe measures turbid broth samples without clarification. The baselines of the sample spectra were therefore not constant (Figure 2). NIR spectral analysis using chemometrics is essential for extracting information from the sample spectral data.

### 2.4. Comparison of PLS Models

A separate calibration model of 162 calibration samples was developed with different spectral preprocessing, and another 30 prediction samples were used for validation purposes. The model generated from the calibration set was tested using the prediction set. The calibration and prediction results of the PLS models of acetic acid, ethanol, TSS, caffeic acid, gallic acid, and tannic acid in the broth samples generated using the entire spectral region are shown in Table 3.

The best model among the conventional PLS models for acetic acid, ethanol, TSS, and gallic acid content was generated without spectral preprocessing. The conventional models yielded lower RMSEP values of 0.419%, 0.500%, 1.057 °Brix and 0.881 µg/mL for acetic acid, ethanol, TSS, and gallic acid, respectively. In contrast, the best PLS models of caffeic acid (RMSEP, 0.877 µg/mL) and tannic acid (RMSEP, 59.15 µg/mL) contents were obtained using SNV- and 2D-preprocessed NIR spectral data, respectively. Our study included light scattering due to interactions between the NIR radiation and sample particles; however, the shift in the absorbance levels due to light scattering may not have affected the linear calibration of the acetic acid, ethanol, TSS, and gallic acid contents. The original NIR absorption spectra of these compounds therefore yielded a better model performance than the PLS models using spectral preprocessing (Table 3). Conversely, the NIR absorptions of caffeic acid and tannic acid probably interfered with the light scattering. Thus, the spectral pretreatment was applied to the spectra before model building to diminish this effect. The results shown in Table 3 reveal that the performance of the caffeic acid and tannic acid models can be improved using the SNV-pretreated spectra and 2D-pretreated spectra, respectively. SNV preprocessing, in which each spectrum was centered and scaled by dividing it by its standard deviation, was introduced to reduce the multiplicative effects of light scattering [32]. The use of 2D preprocessing is also recommended to mitigate the light-scattering effects. Taking the second derivative removes the linear baseline due to scattering, which has negative peaks where the original has a positive peak [32]. After optimizing the spectral preprocessing for each constituent, SCARS calculations were performed on the optimal NIR preprocessing data to identify informative spectral variables for the development of the PLS model.

### 2.5. Spectral Variables Selected by SCARS

**Figure 3:**
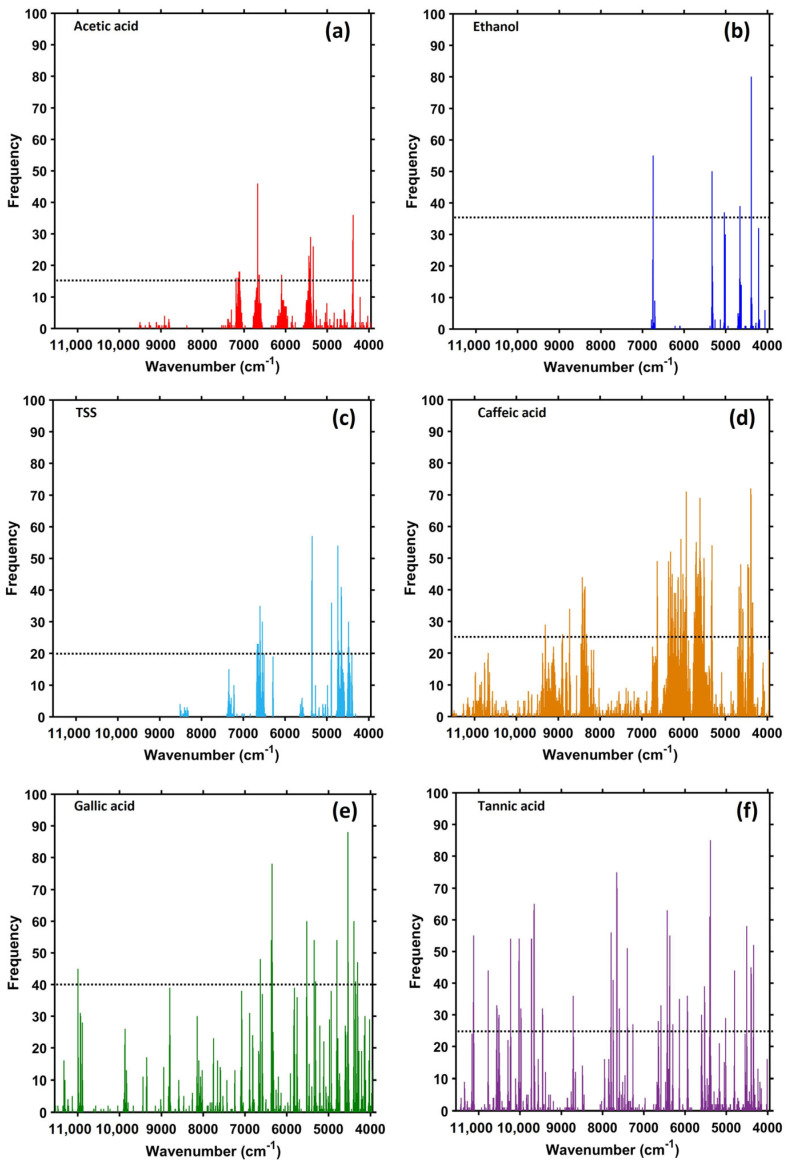
Frequency bar plots of the NIR spectral variables of acetic acid (**a**), ethanol (**b**), TSS (**c**), caffeic acid (**d**), gallic acid (**e**), tannic acid (**f**) and the optimized spectral variables at a selected frequency determined by SCARS (dotted line intersecting the variable bars).

The optimized NIR spectral variables for acetic acid, ethanol, TSS, caffeic acid, gallic acid, and tannic acid obtained using SCARS and their computational parameters (*N*, *M*, frequency level) are reported in Table 4. Fifteen spectral variables (7192, 7144, 7120, 7104, 6672, 6664, 6632, 6096, 5440, 5432, 5408, 5400, 5336, 4384 and 4376 cm^−1^) were selected from the raw spectral data acquired from acetic acid at a frequency of 15 using SCARS (Figure 3a). These variables are similar to those related to acetic acid in vinegar reported by other groups, including Yano et al. [33], who reported 5974 and 5820 cm^−1^ (1674 and 1717 nm) for acetic acid in rice vinegar, and Lui et al. [12], who reported 4253, 4406, and 4527 cm^−1^ for acetic acid in fruit vinegars. The spectral numbers of the variables measured in the current study may not be identical to those reported by previous studies owing to the use of different sample characteristics, spectrometers, and spectral acquisition conditions (resolution, signal-to-noise ratio, interval scanning, etc.). In addition, the present results are in accord with those obtained by Chen et al. [14], who identified the efficient spectral intervals for total acids in commercial vinegars in the regions of 5754.54–6001.39 and 6255.95–6502.79 cm^−1^, and with those of our previous study, in which we observed informative regions in the NIR spectrum of an acetic acid standard (99.85% purity) arising from the first overtones of the COOH and CH stretches in the regions of 7800–7000 cm^−1^ and 6500–5500 cm^−1^, respectively, the OH stretch in the region of 5500–5000 cm^−1^, and the combination band of CH and COOH in 4700–4000 cm^−1^ [34]. All these regions covered the spectral variables of acetic acid identified using SCARS.

Only five spectral variables of (6744, 5328, 5032, 4656 and 4384 cm^−1^) were obtained by SCARS at a frequency of 35 (Table 4 and Figure 3b). These variables were assigned to the OH stretch first overtone, COH second overtone, OH combination, OH deformation, and combination of CH stretching and CH_2_ deformation, respectively [29]. In this case, SCARS identified five informative wavenumbers relevant to the ethanol content from amongst 949 wavenumbers in the entire region. Previous studies investigating the ethanol content during vinegar or vinegar fermentation have not mentioned the specified wavelengths or bands arising from the presence of ethanol (Table 1). These studies have mainly focused on the quantification and band assignment of total acids or other acids, which are the main characteristics of vinegar; however, several studies have identified NIR absorption bands corresponding to the ethanol content in wine; this is similar to our results. Dampers et al. [35] and Cozzolino et al. [9,36] reported absorption bands arising from ethanol production during wine fermentation in the spectral regions at approximately 6060–5715 and 4545–4350 cm^−1^. The former was due to the CH stretch first overtone, whereas the latter was assigned to a combination of the CH stretching and CH deformation of ethanol.

SCARS identified an optimal spectral variable subset for TSS at a frequency of 20 consisting of 6672, 6664, 6648, 6640, 6600, 6592, 6544, 6504, 5360, 5352, 4888, 4736, 4728, 4712, 4656, 4648, 4640, 4488, 4480, 4472 and 4400 cm^−1^ (Table 4 and Figure 3c). Figure 3c shows the characteristics of the variable frequency, where the most informative variables are located in proximity to the first overtone and combination regions in the NIR absorption spectra. Generally, the sugar contents are the total soluble solids expressed by means of TSS value. Pineapple juice was used as the substrate for vinegar fermentation and was a sugar source in the broth samples. In addition, the oxidation products related to sugars, such as sugar alcohols and acidic sugars, were excreted from the cell and dissolved in vinegar during the acetic acid fermentation; this may be included in absorption for TSS [16,23,37]. SCARS yielded 21 spectral variables attributed to the functional groups in the OH stretch first overtone (7000 to 6250 cm^−1^), CH stretch first overtone (5900 to 5500 cm^−1^), OH stretch and CO stretch combinations (5000 to 4750 cm^−1^), CONH combination (4700 to 4650 cm^−1^), and CH combinations of stretching and deformation (4504 to 4250 cm^−1^) [29,30].

The optimized spectral variables of the three phenolic compounds, namely caffeic acid, gallic acid, and tannic acid, suggested by SCARS were 88, 15, and 48 numbers, respectively (Table 4). The SCARS-derived variable frequency characteristics of each phenolic compound (Figure 3d–f) showed that the development of the PLS model required diffuse spectral information from the third, second, and first overtones, including the combination regions in the NIR absorption spectra. Caffeic and tannic acids required more spectral variables than gallic acid to develop a viable model. This may be due to the instability of these acids under acidic conditions, particularly in the case of tannic acid (C_75_H_52_O_46_), which has a complex structure composed of 9-gallic acid and 1-glucose. All forms were present in the broth samples during acetic acid fermentation; therefore, models of caffeic acid and tannic acid involve a large number of concentration-dependent spectral variables. Ríos-Reina et al. [30] observed two low-intensity bands at about 8300 and 5600 cm^−1^ in the vinegar spectrum involving several compounds, including aromatic phenolic compounds. The present study also identified optimal spectral variables for the three phenolic compounds in the same region. In addition to previous reports, SCARS included the aromatic CH, aliphatic CH, CH_2_, CH_3_ of the third overtone (11,530 to 10,000 cm^−1^), aromatic CH and its combination with aliphatic CH (9800 to 8300 cm^−1^), CO carboxylic, CO acid and ester of the second overtone (5400 to 5200 cm^−1^), combined OH and CO stretching (5000 to 4750 cm^−1^), combined alkene CH, aliphatic CH, and CO (4650 to 4520 cm^−1^), and combined CH stretching and deformation and aromatic CH (4504 to 4150 cm^−1^) [29].

### 2.6. Comparison of PLS and SCARS–PLS Models

The statistical results of the SCARS–PLS models constructed using the selected informative spectral variables identified by SCARS were compared with those obtained from the conventional PLS models constructed using the entire spectral region (Table 5). An acceptable model should have a high coefficient of determination (*R_c_*^2^) and a low root-mean-square error of prediction (RMSEP). In all cases, SCARS significantly improved the performance of the calibration model with lower RMSEP values and fewer variables (5 to 88) compared to that obtained by the PLS models using the full spectral region (935 or 949 variables).

In order to prove the accuracy of the statistical results in this study, the performance evaluation of the NIR model was undertaken according to ISO 12099:2017 [22]. The standard error of prediction (SEP) and bias obtained from the SCARS–PLS and PLS models were checked and compared with their confidence limits for unexplained error (*T_UE_*) and confidence limits for bias (*T_b_*), respectively, as shown in Table 6. These criteria could be used to evaluate the accepted model performance if the values obtained for the SEP and bias were within the confidence limits (SEP < *T_UE_*; bias < ± *T_b_*). It can be seen from Table 6 that all statistics obtained from the SCARS–PLS models for acetic acid, ethanol, caffeic acid and gallic acid were acceptable based on these criteria. The result interpretations are that the SEP value was low enough to make it practically acceptable when it was lower than the calculated *T_UE_* value, and that the bias value was not significantly different from zero when it was lower than that calculated ±*T_b_*. Moreover, these SCARS–PLS models provided accurate predictions that were assured with the lowest SEP and bias values. Otherwise, only one PLS model for acetic acid determination could pass the criteria (Table 6). A higher number of acceptable statistical results was obtained from the SCARS–PLS models. This was due to the fact that the informative NIR spectral variables selected by SCARS were strongly correlated with the concentrations of the target analytes in the fermented broths of pineapple vinegar. Therefore, the performance of the obtained SCARS–PLS models was superior to that of the conventional PLS models, which include collinear and unrelated variables. The results found that the conventional PLS did not always yield good results when applied for the NIR analysis of the fermented broths during vinegar fermentation. Meanwhile, the SCARS method was required to improve the model performance. The SCARS–PLS model was developed from a small number of selected spectral variables for specific constituents under optimization and stability testing using SCARS calculations. Therefore, the use of a small number of variables enabled faster measurements, ensuring a rapid, continuous, and repeatable analysis that facilitated the real-time monitoring of the fermentation process.

**Figure 4:**
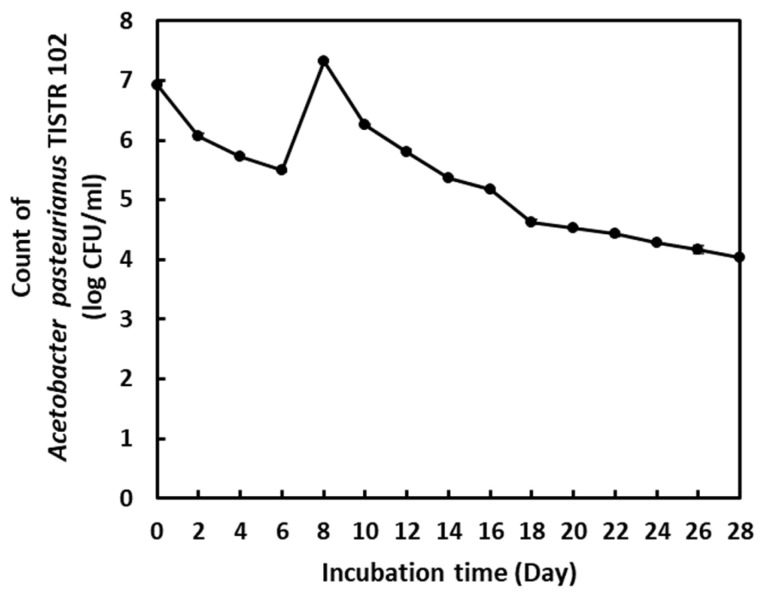
The change in the bacterial population of pineapple vinegar caused by *A. aceti* TISTR 102.

From Table 6, it can be seen that the obtained statistics from the SCARS–PLS models for the TSS and tannic acid determinations were over both confidence limits. The error in the prediction of the TSS and tannic acid contents in the fermented vinegar samples might have occurred due to *A. aceti* bacteria under fermentation conditions. Krepelka et al. [38] investigated the bacteria pattern in the NIR spectrum and reported the NIR spectra of bacteria cells on a glass filter without water absorption effects. According to their results, the NIR spectrum of pure bacteria revealed the vibration of NH (1st overtone) at 7150–6250 cm^−1^, vibrations of the CH and CONH groups (1st overtone) at 5910–5500 cm^−1^, and the vibration of the functional groups related to lipids and proteins at 5250–4000 cm^−1^ [28,38]. These bands in the NIR spectrum displayed functional group oscillations corresponding to the lipids and proteins contained in the cell membranes of bacteria. It was found that different bacteria exhibited NIR absorption bands at the same location [38]. Accordingly, the *A. aceti* bacteria used in this study could have a similar NIR spectrum pattern to that found in their report. According to our results shown in Figure 4, the populations of *A. aceti* TISTR 102 changed through biological activities during pineapple vinegar fermentation. Therefore, *A. aceti* bacteria might have interfered with the NIR absorption of sugars and tannic acid in the samples. However, the SCARS–PLS models for the determination of the TSS and tannic acid contents exhibited a good correlation and should be used for a very rough screening of their changes in vinegar fermentation.

Compared to previous research, Phanomsophon et al. [15] used NIR spectroscopy with full spectral data from 12,500–4000 cm^−1^ to predict the acetic acid and ethanol contents in the rice vinegar internal venturi injector bioreactor. To validate the models for acetic acid and ethanol, the cross-validation method was employed using internal samples, and RMSECV values of 0.244% and 0.273% were obtained, respectively. The SCARS–PLS models used for acetic acid and ethanol determination during pineapple vinegar fermentation in this study obtained better statistical results, with RMSEP values of 0.137% and 0.178% for acetic acid and ethanol predictions, respectively. The number of wavenumber variables used for the SCARS–PLS model building was also largely reduced to 15 for the acetic acid model and to 5 for the ethanol model (Table 5). This may be the advantage of using the optimized informative spectral variables obtained by SCARS to improve the accurate results of the conventional PLS model.

Another previous study was conducted by Liu et al. [13], who reported an RMSEP of 0.035% for the prediction of the acetic acid content in commercial vinegar from apple, lemon and peach using NIR spectroscopy in the range of 7800–4000 cm^−1^. This study also determined the value of soluble solids in commercial rice vinegar and found an RMSEP of 0.189 °Brix by using a portable Vis/NIR spectrometer in the range of 550–1000 nm [12]. Sample measurements were conducted using a cuvette cell. Both RMSEP results for the prediction of acetic acid and soluble solids in commercial vinegars reported by Li et al. [12,13] were lower than those obtained with the SCARS–PLS models in this study (0.137%, acetic acid and 0.875 °Brix, TSS). This is because they used commercial samples, in which these products are usually clear after filtration treatment and the removal of insoluble particles from the liquid, and in which the population of live microorganisms in the finished product is constant. Therefore, their experiments were not influenced by light scattering and changes in the microbial population or sample activity. These results support our conclusion determining that light scattering affects the variation in spectral intensity and that it could not be eliminated during spectral acquisition using the NIR fiber-optic probe without removing particles from the fermented broth vinegar samples. It is noted that no previous study has reported the quantification of caffeic acid, gallic acid and tannic acid in vinegar using NIR spectroscopy.

## 3. Materials and Methods

### 3.1. Sample Preparation

#### 3.1.1. Preparation of Pineapple Juice

Low-grade ripe and overripe pineapples (*Ananas comosus* L. Merr cv. Patavia) were purchased from a wholesale fruit market (Talaad Thai, Khlong Luang District, Pathum Thani Province, Thailand). The pineapples were cleaned, peeled, and crushed to obtain the juice.

#### 3.1.2. Pineapple Wine Fermentation

Yeast culture was firstly prepared by using *Saccharomyces cerevisiae* var. Burgundy. It was obtained from the Institute of Food Research and Product Development (IFRPD), Kasetsart University, Thailand. Yeast strains were activated on yeast extract peptone dextrose (YEPD) agar for 24 to 48 h before use. Then, an inoculum of 5% (*v*/*v*) was prepared by mixing pineapple juice with yeast colonies and incubated for 24 h as a starter (~1 × 10^5^ CFU/mL). Next, pineapple juice and water were mixed at a ratio of 2:1. The initial sugar concentration of the juice was adjusted to 25 °Brix by adding sucrose. Potassium metabisulfite (K_2_S_2_O_5_) was then added to ensure decontamination and to achieve a final concentration of 75 to 100 mg/L. Then, it was transferred to a fermentation tank and mixed with a starter of inoculum yeast cultures (5% *v*/*v*) at the working volume of 15 L. Fermentation was conducted for 10 days at 30 °C to obtain pineapple wine with a 10% *v*/*v* ethanol content. This proper fermentation process for pineapple wine used in this study was obtained from our previous study [39]. Thereafter, the alcohol fermentation was stopped and the pineapple wine was stored at 4 °C to be used as a raw material for pineapple vinegar fermentation.

#### 3.1.3. Pineapple Vinegar Fermentation

*Acetobacter pasteurianus* TISTR 102 starter culture was purchased from the Thailand Institute of Scientific and Technological Research (TISTR). The culture was prepared in sterilized pineapple juice (90 mL) with an initial sugar concentration of 5 °Brix, 95% ethanol (3 mL) and *A. aceti* TISTR 102 (7 mL), and incubated at 30 °C for 72 h before use. The acetification fermentation was performed in an 18 L fermentation tank containing a working volume of 15 L and was started by mixing sterilized pineapple juice, pineapple wine (10% ethanol), and starter culture (~1 × 10^7^ CFU/mL) at a ratio of 6:3:1 and incubating it at 30 °C. After incubation for approximately six days, the ethanol content was low, and the pineapple wine (1 L) was added to the fermentation broth and incubation was continued for 28 days. Aliquots (15–30 mL) of the fermentation broth were collected daily for chemical analyses.

### 3.2. Reference Methods for Quantitative Analysis of the Target Constituents in Fermented Broth of Pineapple Vinegar

Acetic acid, ethanol, TSS, and the phenolic compounds present in pineapple vinegar products were quantified during vinegar fermentation and employed as reference chemical data for the development of an NIR model. After acquiring the NIR spectra, the samples were collected and centrifuged at 6000 RPM using a centrifuge (SC-8, BOECO, Hamburg, Germany), and the supernatants were used for reference analysis in the following procedure:

(1) Analysis of acetic acid content

The acetic acid content was determined using an HPLC apparatus (Shimadzu LC-20A, Tokyo, Japan) with a Bio-Rad Aminex HPX-87H column (300 × 7.8 mm Bio-Rad Laboratories Inc., Hercules, CA, USA) and a Shimadzu RID-UV detector operating at a wavelength of 210 nm. The mobile phase consisted of H_2_SO_4_ (5 mM) at a 0.6 mL/min flow rate, at 60 °C. Samples were filtered through a 0.25 mm microporous membrane filter prior to HPLC analysis. A standard solution of acetic acid with 99.8% purity (Sigma-Aldrich, St. Louis, MO, USA) was prepared to establish the HPLC calibration curve.

(2) Analysis of ethanol content

Gas chromatography (Chromosorb-103, GC4000; GL Sciences; Tokyo, Japan) was performed using an HP5 capillary (30 m × 0.32 mm × 0.25 μm; JW Scientific; Santa Clara, CA, USA) and FID detector under the following conditions: split flow, 50 mL/min; air flow, 250 mL/min; N_2_ carrier flow, 30 mL/min; column temperature, 185 °C; injector temperature, 250 °C; detector temperature, 250 °C. n-Propanol was used as the internal standard [40].

(3) Analysis of the TSS

The TSS concentration in the samples was determined using a digital refractometer (PAL-1, ATAGO, Tokyo, Japan) with a range of 0.0–53.0 °Brix and an accuracy of ±0.2 °Brix.

(4) Analysis of phenolic compounds

The contents of gallic acid, tannic acid, caffeic acid, catechin, coumaric acid, ferulic acid, and rutin, which are reportedly found in pineapples [8,25], were examined in the pineapple vinegar sample. Gallic acid, caffeic acid, catechin, coumaric, ferulic acid and rutin were analyzed using an HPLC apparatus (Shimadzu, Nexera LC-40 series) with a GL Sciences InertSustain C18 column (5 μm, 4.6 × 250 mm) and a Photodiode Array detector. Only gallic acid and caffeic acid standard peaks corresponded to the sample peaks; therefore, the gallic acid and caffeic acid concentrations were quantified under the following conditions: a mobile phase of 1% acetic acid and acetonitrile with a gradient elution program, a flow rate of 0.7 mL/min, a temperature of 30 °C, and a wavelength of 272 nm [41]. The samples were filtered through a 0.45 micron syringe filter nylon membrane prior to HPLC analysis. Standard solutions of gallic acid and caffeic acid (HPLC grade, Biopurify, Chengdu, China) in methanol (3.125, 6.25, 12.5, 25 and 50 μg/mL) were prepared to obtain the HPLC standard curve.

The tannic acid content was determined using a standard curve determined using five tannic acid concentrations [42]. The volume was adjusted to 25 mL with 95% ethanol. The absorbance of each standard concentration was measured using UV–VIS spectroscopy at a wavelength of 280 nm. The tannic acid content in the pineapple vinegar sample was analyzed by mixing an aliquot of the sample (0.05 mL) diluted with 95% ethanol (5 mL) and shaking well. The total tannic acid content of the diluted samples was then analyzed using UV–VIS spectroscopy at 280 nm and compared with the tannic acid standard curve (ACS reagent, Sigma-Aldrich, Beijing, China).

(5) Reference method validation

Method validation using calibration studies and precision testing was adopted from the ICH Harmonised Tripartite Guideline, Validation of Analytical Procedures: Text and Methodology Q2 (R1); (2005) [43]. The calibration studies in the reference method for acetic acid, ethanol, caffeic acid, gallic acid and tannic acid were performed to evaluate the goodness-of-fit of the calibration curves used in the study. Standard solutions of each analyte were analyzed in triplicate (*n* = 3) at 5 different concentrations to determine the coefficient of determination (*R*^2^), limit of detection (LOD) and limit of quantification (LOQ). The precision of the method was tested using replicate analysis (*n* = 7) at a standard concentration above the LOQs (2000 µg/mL acetic acid; 5% ethanol; 12.5 µg/mL caffeic acid; 12.5 µg/mL gallic acid; 2 µg/mL tannic acid). Then, the percent relative standard deviation (%RSD) was calculated using the replicate results obtained. The validation criteria are that *R*^2^ should not be less than 0.9950 and %RSD values should not exceed 2%. The method validation results were found to be acceptable for all methods and are summarized in Table 7.

### 3.3. NIR Fiber-Optic Probe Measurement

FT–NIR measurements of the non-pretreated sample were obtained using a fiber-optic probe (IN271P-02, Bruker Optikcs GmbH & Co. KG, Ettlingen, Germany) in order to collect the NIR transflectance spectral data; this was performed by immersing the optic probe into the fermentation broths at an incubation temperature of 30 °C. The NIR fiber-optic probe consisted of seven fibers in a stainless-steel probe housing measuring 14 cm, a sapphire window with a fixed slit of 1 mm, and an optical path length of 2 mm. The probe was connected to a FT–NIR spectrophotometer (MPA II, Multi-Purpose Analyzer; Bruker Optiks GmbH & Co. KG, Ettlingen, Germany) for spectral acquisition between 11,536 and 3956 cm^−1^ at a spectral resolution of 16 cm^−1^ and 32 scans. Air spectra were used as background. All spectral data were analyzed using the OPUS software (version 8.2: MPA II system, Bruker Optiks GmbH & Co. KG, Ettlingen, Germany) and converted into JCAMP files for multivariate data analysis using the Unscrambler software (version 9.8; CAMO AS, Trondheim, Norway).

### 3.4. Calibration and Prediction Samples

The first fermented broth sample was scanned immediately after mixing and incubating all ingredients. Subsequent sample scans were performed three times a day with an interval of 3 h for 28 days for NIR measurements. These samples were subjected to chemical analysis in parallel. Variations in light scattering were included in the obtained sample spectra because of the characteristic brown and murky colors of the fermented pineapple vinegar broth. Two fermentation batches of pineapple vinegar were prepared for the calibration set of 162 samples used to develop the models. Concurrently, another batch of the same fermentation was separately prepared in a 10 L fermenter containing a 3 L working volume to produce a prediction set of 30 samples to qualify the performance of the model.

### 3.5. Model Development by PLS

A calibration model was developed using NIR spectral variables from the entire region according to the conventional partial least squares (PLS) method. In the first step, PLS calibration models with all NIR wavenumber data from 11,536 to 3956 cm^−1^ were constructed using Unscrambler software to process the spectral data with different preprocessing methods: (1) original spectrum, (2) 2D based on the Savitzky–Golay model (polynomial order of 2, 7 smoothing points), and (3) SNV spectrum. The full cross-validation method was used to determine the optimum number of LVs for PLS by considering the number at which the lowest root-mean-square error of the cross-validation was obtained.

### 3.6. Model Development by SCARS–PLS

The spectral data with a preprocessing method that provided the best performance for the conventional PLS model of each constituent were used as the input for the SCARS calculations. SCARS is a wavelength selection method, first described by Zheng et al. [21], that aims to select important NIR spectral variables to improve the performance of PLS calibration models. The capabilities of the SCARS method have been assessed in numerous studies under different conditions and environments since it was established [44,45,46].

The SCARS algorithm was discussed and detailed by Zheng et al. [21]. SCARS was performed ***N*** times (calculation loop number) to set *N* subsets of variables by removing variables using a stepwise process via enforced wavelength selection and an adaptive reweighted sampling method. The primary subset was the original whole NIR spectra of the calibration samples. The number of repeated sampling times *M* (Monte Carlo sampling) was set before programming; this was associated with each variable subset for stability computation. The stability is defined by Equation (1):

(1) $$c_{j}=\left|\frac{{\bar{b}}_{j}}{S\left(b_{j}\right)}\right|$$

where $c_{j}$ is the stability of the *j*th variable in *M* sampling runs; $b_{j}$ is the mean value of the *j*th variable in *M* sampling runs; and $s(b_{j})$ is the standard deviation of the *j*th variable in *M* sampling runs. Only a positive value of $c_{j}$ according to the absolute value is used and compared.

*N* (number of iterations) and *M* (number of samplings for computing stability with a fixed sampling ratio of 0.6) were set to 20, 50, 100, 200, and 500 for each *N* and *M* combination during the optimized calculations. The calculations were performed while building a series of PLS models to relate the spectral variables to the analyte concentrations. The SCARS algorithm was repeated 30 times for each combination. The optimized *N* and *M* values were defined as those with the lowest mRMSECV values. Subsequently, 100 SCARS calculations were performed with optimized *N* and *M* values, yielding several important subsets of NIR variables to build a PLS model and calculate its RMSECV. The NIR variable subset with the minimum RMSECV was defined as the optimized subset and its variables were considered the most important NIR variables with high stability. A frequency diagram was then generated with the 100 optimized subsets of the NIR variables, and the variables with significant frequencies were extracted into new subsets of variables. PLS models were constructed using these new subsets with different frequencies and validated using a prediction set similar to that of the conventional PLS model. Thereafter, a subset of the optimal variables consisting of informative NIR spectral variables at the same frequency level was selected by considering the subset with the smallest RMSEP value. The SCARS calculation was performed using programs written and in-house coded by Zheng et al. [21] using MATLAB software (version 2020b: The MathWorks Inc., Natick, MA, USA).

### 3.7. Model Performance Evaluation

The performances of the established NIR calibrations using the conventional PLS and SCARS–PLS models were further validated using an independent prediction set. The optimized NIR model is expected to have a high *R_c_*^2^ and low RMSEP values. These evaluation parameters are defined by Equations (2) and (3):

(2) $${R_{c}}^{2}=\frac{{\left(\sum_{i=1}^{n}\left(x_{i}-\bar{x}\right)\left(y_{i}-\bar{y}\right)\right)}^{2}}{\sum_{i=1}^{n}{\left(x_{i}-\bar{x}\right)}^{2}\sum_{i=1}^{n}{\left(y_{i}-\bar{y}\right)}^{2}}$$

(3) $$\mathrm{RMSEP}=\sqrt{\frac{1}{n_{p}}\sum_{i=1}^{n_{p}}(x_{i}-y_{i}{)}^{2}}$$

where $x_{i}$ and $\bar{x}$ are the reference value of sample *i* and the average of the reference values of the samples, respectively; $y_{i}$ is the predicted value of sample *i,* and $\bar{y}$ is the average of the predicted values of the samples; and *n_p_* denotes the number of samples in the prediction set. *R*^2^ reveals the proportion of variance in the NIR-predicted results that can be predicted using the obtained NIR spectral information [28]. RMSEP measures the capacity of the model by expressing the error between the NIR-predicted value and reference values in the prediction set [28].

In order to observe the performance of the best SCARS–PLS and PLS models, the accuracy of all selected models was verified using values of the unexplained error confidence limits (*T_UE_*) and the bias confidence limits (*T_b_*), following the guidelines for determining the NIR spectroscopy of constituents described in ISO 12099:2017 [37]. This International Standard focuses on the validation of the NIR calibration model with an independent validation set. The calculations of *T_UE_* and Tb are performed according to Equations (4) and (5):

(4) $$T_{UE}=SEC\sqrt{F_{\left(\alpha ,\nu ,Μ\right)}}$$

where α (0.05) is the probability of making a type I error; *F* is the appropriate *F*-value for an *F*-test with degrees of freedom associated with SEP ($\nu =n_{p}-1$); *n_p_* is the sample number in a prediction set, SEC (Μ=n−LVs−1); *n* is the sample number in a calibration set; and *LVs* is the latent variable number of the PLS calibration model, and the selected probability of a type I error,

(5) $$T_{b}=\pm \frac{t_{\left(1-\alpha /2\right)}\mathrm{SEP}}{\sqrt{n_{p}}}$$

where *t* is the appropriate Student *t*-value for a two-tailed test with degrees of freedom associated with SEP; and *n_p_* is the sample number in a prediction set, and the selected probability of a type I error (*α* = 0.05).

The standard errors of calibration (SEC), prediction (SEP) and bias can be defined by Equations (6), (7) and (8), respectively.

(6) $$\mathrm{SEC}=\sqrt{\frac{1}{n_{c}}\sum_{i=1}^{n_{c}}(x_{i}-y_{i}-\mathrm{bias}{)}^{2}}$$

(7) $$\mathrm{SEP}=\sqrt{\frac{1}{\left(n_{p}-1\right)}\sum_{i=1}^{n_{p}}(x_{i}-y_{i}-\mathrm{bias}{)}^{2}}$$

(8) $$\mathrm{bias}=\frac{1}{n}\sum_{i=1}^{n}\left(x_{i}-y_{i}\right)$$

SEP indicates the accuracy of the NIR results corrected for the bias between NIR and the reference methods. If SEP is less than *T_UE_*, SEP can be accepted. The significance of the bias is verified using a *t*-test, and the calculation of the bias confidence limits (*T_b_*) determines the limits for the acceptance or non-acceptance of the model performance on the independent prediction set. If the value of the bias is less than *T_b_*, the bias is not significantly different from zero.

## 4. Conclusions

The predictive power of PLS calibration models for determining the content of acetic acid, ethanol, TSS, caffeic acid, gallic acid, and tannic acid in broth samples during pineapple vinegar fermentation was improved by combining them with the calculation of SCARS. The present study demonstrated the efficiency of SCARS, in which a small number of optimized and stabilized spectral variables were discovered in the NIR spectra of each analyte and used in model development. SCARS–PLS models for acetic acid, ethanol, caffeic acid and gallic acid analysis achieved a high performance with accepted accuracy. Nevertheless, the performance of the SCARS–PLS models for the prediction of TSS and tannic acid was not sufficiently accurate based on the verification according to ISO 12099:2017. Therefore, the performance of further studies in order to improve their accuracy by reducing interference in the NIR absorption of TSS and tannic acid in the sample, using orthogonal signal correction before SCARS calculations, is recommended for future research. The present findings confirm that this technique could be applied in the simultaneous monitoring of vinegar fermentation to ensure the quality of fermentation and the products produced according to regulatory requirements.

## Figures and Tables

**Table 1:** Literature reviews on the applications of NIR spectroscopy for the quantitative analysis of the vinegar product or samples produced by acetic fermentation in vinegar production.

Sample	Instrument/ Spectral Region/Measurement Mode	Sample Cell	Chemometric Method	Quantitative Result
Aromatic vinegar (*n* = 120) [18]	FT–NIR spectrometer/ 10,000–4000 cm^−1^/Transmission mode	A standard glass colorimetric ware	PLS	RMSEP 0.3310 mg/mL lactic acid 0.0557 mg/mL malic acid 0.0062 mg/mL L-pyroglutamic acid
Chinese vinegar (*n* = 160) [19]	FT–NIR spectrometer/ 10,000–4000 cm^−1^/Transmission mode	Glass tube 5 mm	Synergy Interval (Si)–PLS	RMSEP 0.26 g/100 mL total acids 1.93 g/100 mL soluble salt-free solids
Fermentation broth of mulberry vinegar [11]	A digital Micro-Mirror-based NIR spectrometer/900–1700 nm/Transmission mode	Cuvette	PLS	RMSEP 0.22% *v/v* total acids 8.11 mg GAE/L total polyphenol
Fruit vinegars (*n* = 180) [12] (apple, lemon, and peach vinegars)	FT–IR–NIR spectrometer/ 7800–4000 cm^−1^/Transmission mode	Liquid cell 1 mm	Least Squares–Support Vector Machine (LS–SVM)	RMSEP 0.35 g/L acetic acid 0.19 g/L tartaric acid 0.17 g/L formic acid 0.0842 pH
Rice vinegar (*n* = 325) [13]	A handheld Vis/NIR spectrometer/ 550–1000 nm/Transmission mode	Cuvette 2 mm	Effective wavelengths–LS–SVM	RMSEP 0.189 °Brix soluble solids 0.008 pH
Rice vinegar (*n* = 150) [15]	FT–NIR spectrometer/ 12,500–4000 cm^−1^/Transflectance mode	0.1 mm glass vial with an aluminum reflector	PLS	RMSECV 2.44 g/L acetic acid 2.73 g/L ethanol
Vinegar sold in China (*n* = 120) [14] (mature, aromatic, and rice vinegars)	FT–NIR spectrometer/ 10,000–4000 cm^−1^/Transmission mode	Quartz cuvette 5 mm	Si–extreme learning machine (ELM)	RMSEP: 0.25 g/100 mL total acids
Vinegar on the market made from different raw materials (*n* = 95) [20]	FT–NIR spectrometer/ 10,000–4000 cm^−1^/Transmission mode	A standard glass colorimetric ware	PLS	RMSEP 0.32 g/mL total acids
Wine vinegar (*n* = 64) [16]	NIR spectrometer/1100–2500 nm/Transflection mode	Quartz liquid cell 2 mm	PLS	Prediction errors ranged 0.008% to 1.15%. Total, non-volatile, and volatile acids; chloride; solids; ash; L-proline; L(+)-tartaric acid; L(−)-malic acid; lactic acid; acetic acid; citric acid; succinic acid; D-malic acid
Wine vinegar (*n* = 107) [17]	Vis/NIR spectrometer/ 400–2500 nm/Transflectance mode	Gold circular reflector cup 0.1 mm	PLS	SEP 3.23 g/L volumic mass 13.97 g/L reducing sugars 1.42 g/100 mL total acidity 0.22 pH

*n* = sample number; RMSECV = root-mean-square error of cross-validation; RMSEP = root-mean-square error of prediction; SEP = Standard error of prediction; °Brix = degree Brix.

**Table 2:** Statistical contents of acetic acid, ethanol, TSS, caffeic acid, gallic acid and tannic acid in the calibration set and prediction set of fermented pineapple vinegar determined using the reference methods.

Analyte	Sample Set	Range	Mean	SD	*n*
Acetic acid (%*w/v*)	Calibration set	4.69 × 10^−2^–4.24	1.27	1.12	162
	Prediction set	5.00 × 10^−2^–4.19	1.67	1.29	30
Ethanol (%*v/v*)	Calibration set	5.00 × 10^−3^–7.00	3.68	2.17	162
	Prediction set	2.10 × 10^−2^–4.76	2.12	1.46	30
TSS (°Brix)	Calibration set	7.90–10.80	9.65	0.68	161 ^a^
	Prediction set	7.97–9.80	9.07	0.63	30
Caffeic acid (µg/mL)	Calibration set	1.23–7.46	3.78	1.64	162
	Prediction set	1.63–6.85	4.69	1.75	30
Gallic acid (µg/mL)	Calibration set	3.46–5.98	4.90	0.66	162
	Prediction set	3.99–5.35	4.67	0.33	30
Tannic acid (µg/mL)	Calibration set	138.82–288.30	198.09	39.08	162
	Prediction set	144.32–204.69	168.03	19.34	30

SD = Standard deviation; *n* = number of samples; ^a^ number of samples remaining after removing outliers.

**Table 3:** Statistical results of PLS models for acetic acid, ethanol, TSS, caffeic acid, gallic acid and tannic acid in fermented pineapple vinegar.

Analyte	Spectral Preprocessing	LVs	*R_c_* ^2^	RMSEP
Acetic acid (%*w/v*)	None	5	0.870	0.419
	2D	5	0.888	0.509
	SNV	4	0.855	0.532
Ethanol (%*v/v*)	None	6	0.876	0.500
	2D	6	0.974	0.602
	SNV	5	0.969	0.632
TSS (°Brix)	None	9	0.960	1.057
	2D	8	0.956	1.107
	SNV	9	0.947	1.080
Caffeic acid (µg/mL)	None	8	0.846	0.974
	2D	6	0.832	0.914
	SNV	7	0.825	0.877
Gallic acid (µg/mL)	None	10	0.638	0.881
	2D	12	0.755	0.902
	SNV	8	0.567	1.064
Tannic acid (µg/mL)	None	10	0.682	61.48
	2D	10	0.694	59.15
	SNV	9	0.641	66.78

LVs = number of latent variables; *R**_c_***^2^ = coefficient of determination; RMSEP = root-mean-square error of prediction; 2D = second derivatives; SNV = standard normal variate.

**Table 4:** Informative spectral variables selected by SCARS and the optimal parameters for the SCARS calculations for acetic acid, ethanol, TSS, caffeic acid, gallic acid and tannic acid in fermented pineapple vinegar.

Analyte	Selected Informative Spectral Variable by SCARS (cm^−1^)	Optimal SCARS Parameter		
		*N*	*M*	Frequency Level
Acetic acid (%*w/v*)	7192, 7144, 7120, 7104, 6672, 6664, 6632, 6096, 5440, 5432, 5408, 5400, 5336, 4384, 4376	200	20	15
Ethanol (%*v/v*)	6744, 5328, 5032, 4656, 4384	500	50	35
TSS (°Brix)	6672, 6664, 6648, 6640, 6600, 6592, 6544, 6504, 5360, 5352, 4888, 4736, 4728, 4712, 4656, 4648, 4640, 4488, 4480, 4472, 4400	500	200	20
Caffeic acid (µg/mL)	11,520, 9312, 9304, 8896, 8728, 8432, 8424, 8416, 8384, 8376, 8368, 8360, 8328, 6632, 6624, 6368, 6360, 6352, 6320, 6312, 6304, 6280, 6272, 6264, 6232, 6224, 6216, 6192, 6184, 6144, 6136, 6128, 6080, 6072, 6064, 6056, 6024, 6016, 6008, 6000, 5976, 5968, 5960, 5952, 5944, 5936, 5928, 5744, 5720, 5712, 5704, 5696, 5688, 5672, 5664, 5656, 5648, 5640, 5632, 5624, 5616, 5608, 5600, 5592, 5584, 5576, 5568, 5528, 5520, 5512, 5504, 5336, 5328, 5320, 4672, 4664, 4632, 4624, 4584, 4576, 4464, 4448, 4440, 4392, 4384, 4376, 4352, 4344	200	100	25
Gallic acid (µg/mL)	10,992, 6632, 6368, 6360, 6352, 6344, 5520, 5344, 5312, 4800, 4536, 4528, 4392, 4352, 4304	500	100	40
Tannic acid (µg/mL)	11,128, 11,120, 10,768, 10,560, 10,552, 10,512, 10,504, 10,224, 10,024, 10,016, 9976, 9968, 9720, 9664, 9656, 9456, 9448, 8712, 7800, 7792, 7744, 7656, 7648, 7592, 7400, 7392, 7264, 6648, 6584, 6432, 6424, 6376, 6368, 6296, 6136, 5944, 5936, 5600, 5528, 5520, 5400, 5384, 5016, 4800, 4504, 4400, 4392, 4336	200	200	25

*N* = number of iterations; *M* = number of samplings for computing stability with a fixed sampling ratio of 0.6.

**Table 5:** Statistical results of PLS and SCARS–PLS models for acetic acid, ethanol, TSS, caffeic acid, gallic acid and tannic acid in fermented pineapple vinegar.

Analyte	Spectral Preprocessing	Method	Number of Variables	LVs	*R_c_* ^2^	RMSEP
Acetic acid (%)	None	SCARS–PLS	15	4	0.874	0.137
		PLS	949 ^a^	5	0.870	0.419
Ethanol (%)	None	SCARS–PLS	5	5	0.973	0.178
		PLS	949 ^a^	6	0.876	0.500
TSS (°Brix)	None	SCARS–PLS	21	3	0.903	0.875
		PLS	949 ^a^	9	0.960	1.057
Caffeic acid (µg/mL)	SNV	SCARS–PLS	88	8	0.938	0.637
		PLS	949 ^a^	7	0.825	0.877
Gallic acid (µg/mL)	None	SCARS–PLS	15	12	0.752	0.340
		PLS	949 ^a^	10	0.638	0.881
Tannic acid (µg/mL)	2D	SCARS–PLS	48	10	0.891	31.12
		PLS	935 ^b^	10	0.694	59.15

LVs = number of latent variables; *R**_c_***^2^ = coefficient of determination; RMSEP = root-mean-square error of prediction; 2D = second derivatives; SNV = standard normal variate; ^a^ 949 = All variable numbers in the wavelength region of 11,536–3956 cm^−1^ with none or after SNV spectral preprocessing; ^b^ 935 = All variable numbers in the wavelength region of 11,480–4008 cm^−1^ after second derivatives spectral preprocessing.

**Table 6:** Comparison of statistics for assessment of the model performance between PLS and SCARS–PLS models for acetic acid, ethanol, TSS, caffeic acid, gallic acid and tannic acid in fermented pineapple vinegar following ISO 12099:2017.

Best Model	Method	Statistic	Obtained Result	Criterion	Performance
Acetic acid (%)	SCARS–PLS	SEP	0.136	*T_UE_* = 0.480	accepted
		bias	0.023	*T_b_* = ±0.051	accepted
	PLS	SEP	0.424	*T_UE_* = 0.487	accepted
		bias	0.043	*T_b_* = ±0.158	accepted
Ethanol (%)	SCARS–PLS	SEP	0.173	*T_UE_* = 0.426	accepted
		bias	−0.053	*T_b_* = ±0.065	accepted
	PLS	SEP	0.421	*T_UE_* = 0.464	accepted
		bias	−0.275	*T_b_* = ±0.157	not accepted
TSS (°Brix)	SCARS–PLS	SEP	0.662	*T_UE_* = 0.256	not accepted
		bias	−0.586	*T_b_* = ±0.247	not accepted
	PLS	SEP	0.754	*T_UE_* = 0.165	not accepted
		bias	−0.754	*T_b_* = ±0.282	not accepted
Caffeic acid (µg/mL)	SCARS–PLS	SEP	0.630	*T_UE_* = 0.653	accepted
		bias	0.148	*T_b_* = ±0.235	accepted
	PLS	SEP	0.890	*T_UE_* = 0.829	not accepted
		bias	−0.068	*T_b_* = ±0.332	accepted
Gallic acid (µg/mL)	SCARS–PLS	SEP	0.342	*T_UE_* = 0.396	accepted
		bias	−0.049	*T_b_* = ±0.128	accepted
	PLS	SEP	0.615	*T_UE_* = 0.478	not accepted
		bias	−0.641	*T_b_* = ±0.230	not accepted
Tannic acid (µg/mL)	SCARS–PLS	SEP	27.051	*T_UE_* = 15.584	not accepted
		bias	16.163	*T_b_* = ±10.101	not accepted
	PLS	SEP	53.433	*T_UE_* = 26.106	not accepted
		bias	−27.176	*T_b_* = ±19.952	not accepted

*T_UE_* = unexplained error confidence limits (*α* = 0.05); *T_b_* = bias confidence limits (*α* = 0.05).

**Table 7:** Results of reference method validation for HPLC analysis of acetic acid, caffeic acid and gallic acid, GC analysis of ethanol, and UV analysis of tannic acid.

Analytes	Analytical Method	Response	Linear Range	*R* ^2^	LODs	LOQs	%RSD
Acetic acid (μg/mL)	HPLC	Rt = 14.5 min.	100–10,000	0.9993	0.13	0.38	0.48
Ethanol (%)	GC	Rt = 1.853 min (Ethanol) Rt = 3.246 min (n-propanol; internal standard)	0.25–10	0.9986	0.02	0.05	1.01
Caffeic acid (μg/mL)	HPLC	Rt = 14.559 min	3.125–50	1.0000	0.02	0.07	0.11
Gallic acid (μg/mL)	HPLC	Rt = 6.523 min	3.125–50	1.0000	0.03	0.09	0.17
Tannic acid (μg/mL)	UV spectrometry	Abs, 280 nm	2–18	0.9997	0.09	0.28	0.99

LODs = limit of detection; LOQs = limit of quantification; %RSD = percent relative standard deviation; Rt = retention time.

## Data Availability

All data supporting the conclusions of this article are included in the manuscript.
